# Empowering biological knowledgebases: advances in human-in-the-loop AI-driven literature curation

**DOI:** 10.1093/bioadv/vbag028

**Published:** 2026-01-27

**Authors:** Valerie Wood, Matt Jeffryes, Andrew F Green, Matthias Blum, Sandra Orchard, Simona Panni, Federica Quaglia, Raul Rodriguez-Esteban, James Seager, Silvio C E Tosatto, Ulrike Wittig, Melissa Harrison

**Affiliations:** Department of Biochemistry, University of Cambridge, Cambridge CB2 1GA, United Kingdom; Literature Services, European Molecular Biology Laboratory, Wellcome Genome Campus, Hinxton, Cambridgeshire CB10 1SD, United Kingdom; Sequence Family Resources, European Molecular Biology Laboratory, Wellcome Genome Campus, Hinxton, Cambridgeshire CB10 1SD, United Kingdom; Sequence Family Resources, European Molecular Biology Laboratory, Wellcome Genome Campus, Hinxton, Cambridgeshire CB10 1SD, United Kingdom; Protein Function Content, European Molecular Biology Laboratory, Wellcome Genome Campus, Hinxton, Cambridgeshire CB10 1SD, United Kingdom; Department of Biology, Ecology and Earth Science, University of Calabria, Rende, 87036, Italy; Biomedical Sciences, University of Padova, Padova 35131, Italy; Roche Innovation Center Basel, Basel 4070, Switzerland; Translating Biotic Interactions, Rothamsted Research, Harpenden AL5 2JQ, United Kingdom; Biomedical Sciences, University of Padova, Padova 35131, Italy; Scientific Databases and Visualization, Heidelberg Institute for Theoretical Studies, Heidelberg 69118, Germany; Literature Services, European Molecular Biology Laboratory, Wellcome Genome Campus, Hinxton, Cambridgeshire CB10 1SD, United Kingdom

## Abstract

Biological knowledgebases facilitate discovery across the life sciences by structuring experimental findings into human-readable and computable formats. These essential resources are maintained by a small number of professional biocurators worldwide and face combined chronic underfunding and the exponential growth of the literature. In this perspective, we review how artificial intelligence, particularly large language models and agentic systems, can augment literature-curation workflows. Applications include literature recommendation, entity recognition, data extraction, summarization, ontology development, and quality control with emphasis on published use cases at Global Core BioData Resources and ELIXIR Core Data Resources. We identify key challenges, including the scarcity of training data, difficulty in extracting complex relationships, and concerns about error propagation. To address these challenges, we propose a human-in-the-loop framework where generative artificial intelligence approaches accelerate routine tasks while curators provide critical evaluation and domain expertise. We also propose practical recommendations for the community, including the creation of shared benchmark datasets, harmonized evaluation frameworks, and best-practice guidelines for transparent human-in-the-loop AI deployment in biocuration. These synergistic partnerships will be critical to ensure biological rigour, accelerating knowledge integration while maintaining the quality essential for trusted biological resources.

## 1 Introduction

In this perspective, we review how artificial intelligence (AI), particularly large language models (LLMs) and agentic systems, can augment literature-curation workflows for knowledgebases. We first describe the importance of knowledgebases to the scientific ecosystem, the biocuration process and the challenges of scalable curation. Automated workflows are already in development or in use at several curated knowledgebases (Alliance of Genome Resources Consortium 2024, UniProt Consortium 2025). Although our focus is on the implementation of AI technologies into the curation workflow, a brief description of the technologies (pre- and post-LLMs) is provided.

**Figure 1:**
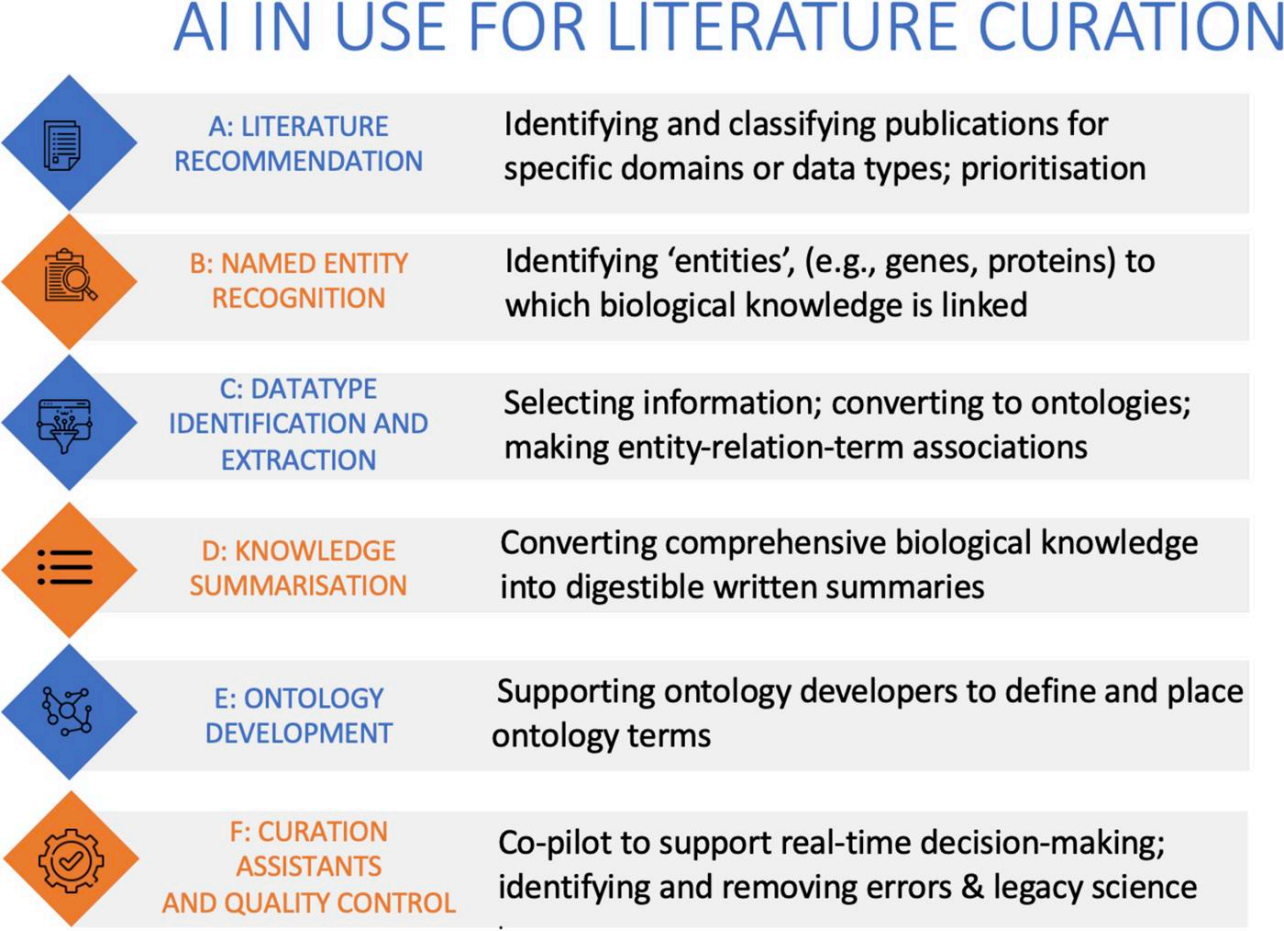
Six key areas of AI-supported literature curation: (A) literature recommendation, (B) named-entity recognition, (C) data type identification and extraction, (D) knowledge summarization, (E) ontology development, and (F) curation assistants and quality control. Each category represents a distinct step in the curation process or a standalone curation task, in which AI models, ranging from ML classifiers to LLMs, are deployed or in development. Examples include triaging relevant publications (A), linking entity mentions to database identifiers (B), identifying curatable data types and their relationships (C), generating concise textual summaries (D), suggesting or validating ontology terms (E), and assisting curators through interactive or agentic systems (F). Together, these components reflect a modular AI ecosystem designed to support, rather than replace, expert biocurators.

We then examine advances in automating routine tasks to improve the efficiency of manual biocuration, allowing curators to focus on activities that require expert judgement. The applications reviewed include named-entity recognition (NER), fact extraction, gene summarization, ontology development, and the use of AI-based tools as curation assistants, with an emphasis on published implementations by knowledgebases with Global Core BioData Resource (GCBR) or ELIXIR Core Data Resource (CDR) status (Fig. 1).

We conclude by summarizing the challenges that tool providers and biocurators must address for AI technologies to significantly increase curation efficiency. These challenges include identifying tasks suitable for assistance or automation, providing and sharing improved training datasets, better evaluation procedures, and addressing concerns about error propagation and its mitigation. Finally, we emphasize the crucial human-in-the-loop role of biocurators in implementing, evaluating, and refining AI tools and their outputs.

### 1.1 Why are biological knowledgebases important?

As biological knowledge increases, researchers are confronted with managing the vast and complex information associated with experimental findings for their biological objects of interest (e.g. genes, proteins, alleles, chemicals, diseases, pathways). By capturing both detailed findings and overarching conclusions, knowledgebases offer precise and comprehensive insights into molecular and cellular entities by describing their biological functions, phenotypes, interactions, and modifications. In this setting, curated biological knowledgebases, which add value by organizing and integrating knowledge, have become increasingly indispensable community tools (Oliver *et al.* 2016, Bellen *et al.* 2021, Lipshitz 2021, Wood *et al.* 2022, Pop *et al.* 2025). Knowledgebases are different from repositories where data are deposited (with associated metadata), although some resources provide both functions.

This perspective is concerned only with knowledgebases that manually and systematically integrate experimental data from the peer-reviewed scientific literature. The content of these meticulously curated resources is maintained by a relatively small group of approximately 100–200 full-time professional biocurators worldwide (de Crécy-Lagard *et al.* 2022). Although the impact of knowledgebases is difficult to quantify, users consistently regard them as indispensable to their research, supporting their implicit role in enabling scientific discovery. For example, in a survey of users of PomBase—the fission yeast knowledgebase—over 92% of users (*n* = 623) responded that the absence of PomBase would cause severe to extreme impacts on their projects, with many indicating that numerous projects would no longer be feasible if PomBase were absent (Rutherford *et al.* 2024). This insight is consistent with broader findings from the 2021 EMBL-EBI impact report, which underscores how EMBL-EBI-managed data resources (including knowledgebases) significantly improve research efficiency and deliver exceptional value relative to their operational costs (Cook *et al.* 2018). The contribution of UniProt to the scientific community and wider economy has also been assessed in a case study, conducted as part of the EU-funded PathOS project (doi.org/10.5281/zenodo.15732022). The analysis estimated that each user gains a net benefit of up to €5475 per year and saves approximately 219 h per year, with the resource providing a total annual benefit of between €373 and 565 million to its community of scientific users.

Knowledgebases play a vital daily role in supporting researchers who both generate experimental data and depend on curated biological information. For these primary users, maintaining the highest possible accuracy is crucial, as curated content directly informs hypothesis generation, experimental design, and the interpretation of results (International Society for Biocuration 2018, Rutherford *et al.* 2024, Pop *et al.* 2025). To meet this need, knowledgebases strive for near-zero error rates in the manual curation of experimental findings. The curated facts are typically encoded in machine-readable ontologies—standardized, interconnected vocabularies that ensure consistency and interoperability across datasets (Smith *et al.* 2007). By combining rigorous curation with formal ontological structures, knowledgebases provide a uniquely reliable foundation for AI applications (Confalonieri *et al.* 2024). Consequently, biological knowledgebases now serve a dual function: they provide authoritative knowledge for hypothesis generation, and they constitute the definitive ground truth for training and benchmarking AI systems in the life sciences domain.

### 1.2 What is literature curation?

Primary literature curation involves reading the full text of peer-reviewed publications to identify the entities to be curated (e.g. genotypes, genes, proteins, cells) and to associate them with biological facts (Poux *et al.* 2014). This task requires an in-depth understanding of the biology described, the standard vocabularies or ontologies used to describe it, and the relative importance of different types of information to the particular user community of the resource.

The term *annotation* has different meanings in the text mining and biocuration communities, which can lead to confusion. In text mining, annotation typically refers to labelling spans of text as entities, such as genes, proteins, or biological concepts, although sophisticated text mining projects are sometimes used to detect relationships between entities. In biocuration, annotation involves manually extracting structured biological information from the literature and integrating it into knowledgebases based on experimental results. While both processes involve assigning labels, text-mined annotation usually includes many labels that are irrelevant to the biocuration task, which focuses only on biologically meaningful *associations* between entities and ontology terms, evidence, and source. Evidence refers to the formal description of how a gene–term association was established, for literature curation this is usually the type of experimental assay and is typically represented using terms from the Evidence and Conclusion Ontology (ECO) (Giglio *et al.* 2019). Provenance (or source) is always a publication for experimental annotation, but could also be additionally supported by a ‘text span’ of the sentence(s) used by a curator to make an assertion. To reduce ambiguity, we use the term *association* throughout this manuscript to refer to ‘structured biocuration annotations’.

**Figure 2:**
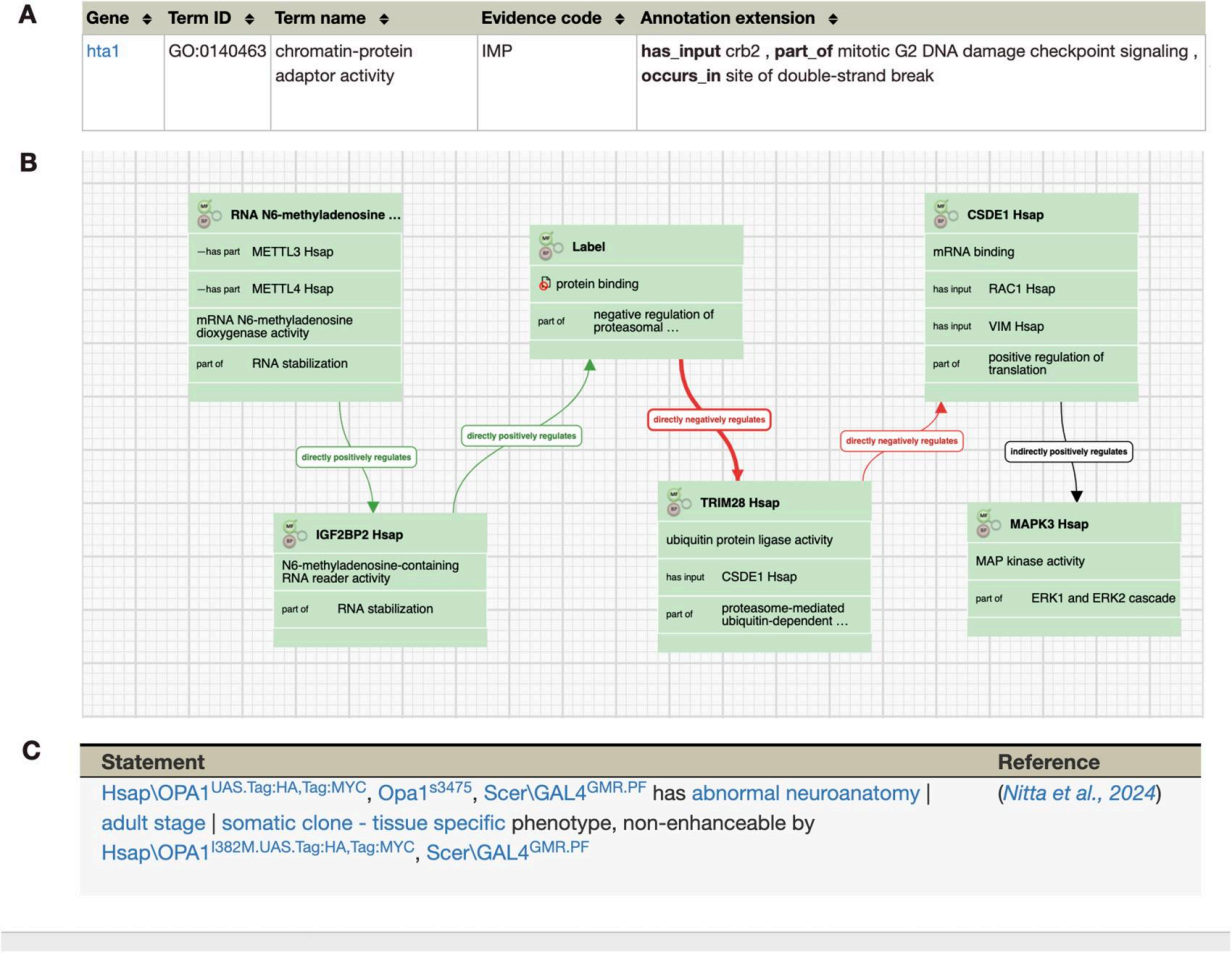
Example entity-to-term associations curated from the literature by knowledgebases. A standard gene ontology molecular function association to the term ‘chromatin-protein adaptor activity’ (GO:0140463) at PomBase connects the histone H2A alpha (hta1) to the protein Crb2 (crb2), the protein recruited by the modified histone during DNA damage checkpoint signalling, using the IMP (‘inferred from mutant phenotype’) evidence. The source (PMID:24806815) is not shown in this part of the curator view because the workflow is publication centric and displaying the PMID on each of the 50 individual annotations for this publication would be redundant. However, publication sources are presented to the end user in PomBase (2A). A collection of molecular functions connected into a pathway using GO-Causal Activity Model (GO-CAM) syntax in the Noctua pathway curation tool (Thomas *et al*. 2019). All GO-CAM associations are supported by evidence and source which are hidden for simplicity in this curation view but are visible to the end user in hosting resources. A single GO-CAM is usually constructed by integrating multiple types of evidence from many publications (2B). A FlyBase phenotype (abnormal neuroanatomy) resulting from genetic interactions between two alleles (1C). All associations include precise entity labels (e.g. genes, alleles, genotypes), evidence, source, ontology term(s), and additional data type-specific information. The individual components of an association also need to be connected by standardized relations.

As biological models become increasingly detailed and interconnected, biocuration efforts focus increasingly on capturing more specific connections between entities and developing knowledge graphs (KGs) or pathway models for entire processes. The examples in Fig. 2A–C illustrate the complexity of entity labels, their associated data, and connections drawn from multiple ontologies at a selection of resources.

### 1.3 The challenge of scalable literature curation

The exponential growth in protein sequences (UniProtKB/TrEMBL Release 2025_03 includes ∼253 million proteins) and the vast volume of publications (∼37 million research article records in Europe PMC; europepmc.org) have long raised concerns about the sustainability of knowledge curation (Baumgartner *et al.* 2007, Bourne *et al.* 2015). However, as demonstrated by Poux *et al*. only a subset of publications contain data curatable by UniProtKB—at least 90% of the papers in PubMed (pubmed.ncbi.nlm.nih.gov) are entirely ‘out of scope’ (i.e. without gene or protein-specific information) for UniProtKB and, after review, a maximum of 2%–3% of the documents indexed were considered suitable for manual curation (Poux *et al.* 2017).

Articles may be considered unsuitable for knowledgebase curation for several reasons. For example, publications can describe the use of an organism as a tool in biotechnology, normal species-level biology, sequencing reports, or evolutionary studies, thus providing no curatable entity-related information that is suitable for existing resources. Curators typically prioritize articles that present novel data or provide specific information to address knowledge gaps. If only 2%–3% of publications are curatable for UniProtKB, it is possible that only 3%–6% of PubMed articles are curatable across all knowledgebases. If so, this would represent 2–3 million publications, increasing annually. Although a substantial proportion of these articles may already be curated, the remaining effort is still considerable (though achievable through appropriate scaling). The magnitude of this challenge underscores the need for computational assistance.

Transferring curated functional data from extensively researched genes in well-studied species to their less-studied homologs in other species (known as *functional transfer* or *function prediction*) entirely depends on the accurate curation of primary literature. The impact of the 1.2 million manually curated, experimentally supported Gene Ontology (GO) associations is substantial. These annotations are used to support over 1 billion additional associations inferred through manual, computational, or phylogenetic transfer methods (source: ebi.ac.uk/QuickGO/annotations, June 2025). Functional transfer to unstudied or unannotated genes relies entirely on manually curated experimental annotations. This makes their accuracy is crucial to prevent the propagation of errors, as demonstrated through the phylogenetic transfer of functional data (Feuermann *et al.* 2025). However, a detailed analysis of function prediction falls outside the scope of this perspective.

### 1.4 Leveraging AI for enhanced biocuration

AI/ML-based tools have long supported biocuration pipelines, particularly in tasks such as entity recognition, relationship extraction, and literature triage. Before 2022, these tools relied on natural language processing (NLP), rule-based systems, dictionary/lookup approaches, and hybrid models. The introduction of transformer-based models, such as BERT, demonstrated the effectiveness of large-scale pretraining for language understanding tasks (Devlin *et al.* 2019). The launch of OpenAI’s ChatGPT in late 2022 marked a further leap in capability. ChatGPT was built upon earlier transformer models (e.g. GPT-3; Brown *et al.* 2020) and incorporated instruction tuning (Ouyang *et al.* 2022), enabling more context-aware and goal-directed language generation.

Since then, LLMs have enabled the development of more flexible, conversational, and context-aware tools for biomedical applications (Fang *et al.* 2023). Retrieval-augmented generation (RAG) architectures combined semantic search (e.g. using vector embeddings from BioLinkBERT or Sentence-BERT-style encoders) with a generative model, allowing the system to provide more accurate answers based on relevant contextual information. This approach enables the model to access published or curated knowledge in real-time, improving accuracy and supporting on-the-spot reasoning. While RAG reduces errors by grounding responses in retrieved information, ensuring that all outputs are consistently correct remains a challenge (Zhou *et al.* 2024).

Emerging agentic AI systems layer goal-oriented planning, autonomous action execution, observation, and adaptation on top of LLMs. In this context, ‘deep research’ harnesses these agentic capabilities to perform extensive, multi-step investigations. Through autonomous planning, tool use, and iterative reasoning, LLM-based systems can function as effective digital research assistants. By maintaining memory and context across tasks, agents can better interpret biological information, resolve ambiguities, and apply curation rules more consistently.

Numerous reviews have already examined the specific technologies we reference (Minaee *et al.* 2025, Zhao *et al.* 2025, and others); in this perspective, we focus on implementations that streamline the curation of published literature for biocuration knowledgebases, particularly LLM-driven applications, concentrating on concrete use cases and outputs rather than model architectures. Likewise, we omit discussion of ML evaluation standards, since these topics are covered elsewhere (Walsh *et al.* 2021).

## 2 Application of AI-based methods to literature curation tasks

### 2.1 Literature recommendation and prioritization

Literature recommendation involves systematically identifying and acquiring topic-specific publications from repositories such as PubMed and Europe PMC. Literature identification and retrieval are fundamental to biocuration, but basic keyword search systems often return suboptimal results (false positives and false negatives). Most knowledgebases require bespoke literature identification strategies customized to their specific scientific domain and focus. Typically, this process begins with a broad search to identify all *potentially relevant* publications, followed by filtering steps to identify *genuinely relevant* publications (The UniProt Consortium 2017, Blake *et al.* 2021). In some cases, such as a resource targeting species-specific content, a simple search can be sufficient to identify most of the broad corpus of interest. For instance, literature acquisition at the Alliance of Genome Resources (AGR) and at PomBase begins with automated, organism-specific PubMed queries to retrieve candidate publications for each model organism database. Other knowledgebases focus on specific data types. They must identify all publications related to those domains, such as reactions for the Rhea reaction database, complexes for ComplexPortal, protein families for Pfam, and cell types for Cellosaurus. Although UniProtKB does not aim to comprehensively identify all potentially in-scope articles, pipelines using tools such as PubTator and LitSuggest are employed for specific data types, and by curators to create search strategies for particular projects (Allot *et al.* 2021, Wei *et al.* 2024).

This broad classification of publications as ‘in-scope’ or ‘out of scope’ is often accompanied by an initial flagging or first-pass review process to further classify documents, commonly referred to as *triage*. This step serves two purposes: firstly, to eliminate false positives (e.g. excluding records relating to the wrong species in an organism-specific database), and, secondly, to flag documents that are ‘in scope’ for a resource but not suitable for curation (e.g. methods papers, review articles, or observations that cannot be associated with a specific entity).

Resources that manage large volumes of literature typically use different teams to curate specific data types, and in this context, data type flagging and prioritization reports become necessary. Each resource faces unique challenges in identifying relevant publications for curation and the types of curatable data contained within them. Below, we summarize key literature resources and open-source tools currently used in production at GCBR/CDR knowledgebases, highlighting their application in literature triage and recommendation.

#### 2.1.1 Resources for literature recommendation

*Europe PMC* (europepmc.org) is an open-access database of life sciences and biomedical literature mirroring PubMed and PubMed Central, supplemented by preprints from 35 additional servers (Rosonovski *et al.* 2024). In addition to the 10 million full-text articles on the Europe PMC website, Europe PMC integrates with Unpaywall to navigate to a further 10 million full-text articles (Piwowar *et al.* 2018). Europe PMC applies text mining to identify approximately 2 billion biological entities. All metadata and value-added content from text mining are exposed through open APIs, enabling bulk analysis and seamless integration into literature-processing and curation workflows.

In a recent release, the Europe PMC text-mining pipeline has incorporated machine learning models to enhance the recall and precision of these annotations (Tirunagari *et al.* 2025). The ‘Data Links’ section on an article landing page provides links to curation by knowledgebases submitted through the ‘External Links’ service or via the EBI Search API (for resources managed by EMBL-EBI). Text-based supplementary files are also text-mined for entities and made available for website search and via APIs. An ‘Article Status Monitor’ tracks article status—including preprint version history, peer review status, withdrawal, and the publication and retraction status of the final journal article. Article status can also be queried via a web form or REST API, enabling integration into automated literature-curation pipelines. The Genome-Wide Association Study (GWAS) catalogue uses this monitoring system to release summary statistics linked to a preprint after publication in a peer-reviewed journal.

*PubTator* (ncbi.nlm.nih.gov/research/pubtator3/) is a web-based text-mining tool integrated with PubMed and used primarily to annotate biomedical literature with tags (Wei *et al.* 2024). The scope includes abstracts from PubMed and full-text articles from PubMed Central (PMC). PubTator is designed for NER and topic mining (discussed below), but can also be used for primary literature classification and triage tasks. PubTator is used by several knowledgebases, including UniProtKB, for literature identification.

*The SIB Literature Services* (SIBiLS, sibils.org) offers customizable information retrieval from MEDLINE, open-access articles in PubMed Central, and a dedicated biodiversity collection. It also includes supplementary data and supports information extraction from images (Gobeill *et al.* 2020). Updated daily, SIBiLS is accessible via a REST API and can be seamlessly integrated into curation workflows. Its content is semantically enriched by mapping biomedical entities using a comprehensive set of vocabularies, encompassing nearly 2 billion mapped terms. Search results are ranked by relevance, enabling efficient literature triage. SIBiLS is actively used for literature curation in resources such as DisProt, a database of intrinsically disordered proteins, and Cellosaurus, a knowledgebase of cell lines.

*LitSuggest* (ncbi.nlm.nih.gov/research/litsuggest) is a web-based literature recommendation tool designed to help curators identify relevant articles in PubMed (Allot *et al.* 2021). It uses ML classifiers built through text mining of a curated list of relevant articles, provided as PubMed identifiers. As users accept or reject suggested papers, the model is continuously refined. LitSuggest can also integrate LLMs to enhance the discovery and prioritization of articles, leveraging both structured data (e.g. citation networks) and unstructured data (e.g. full-text content). LitSuggest is used by UniProtKB, Rhea, the Complex Portal, the National Human Genome Research Institute (NHGRI), and the GWAS Catalogue to identify publications that contain functional information on enzymes, protein complexes, and genomic variation.

*The Alliance of Genome Resources* (AGR) (alliancegenome.org) is developing the Alliance Bibliographic Central (ABC), an integrated literature database, to import publications, run literature classification and triage pipelines, identify entities, and manage the curator workflow centrally for all AGR model organism databases (Alliance of Genome Resources Consortium 2024). Following initial classification, curators at individual resources perform a manual triage to eliminate false-positive results. The AGR will centrally integrate new and existing classification and triage methods for all members. To support literature-curation workflow management within the ABC, the AGR has developed the Alliance Tags for Papers (ATP) ontology—a structured tagging schema for managing publications (github.com/alliance-genome/agr_atp_ontology). The ATP uses tags to categorize publications by type, data, topic, and workflow status (e.g. file availability, assigned to a curator, or curation completed). Because ABC aims to support comprehensive end-to-end article classification—encompassing NER, data extraction, and curator review—the ATP ontology provides a valuable metadata source. This structured tagging schema can help to identify which publications are appropriate for inclusion in, or exclusion from, AI training and evaluation datasets based on their type, content, and curation status.

AI-driven and rule-based text-mining methods are already widely deployed for initial document retrieval, classification, and prioritization across numerous knowledgebases, and there is an ongoing shift towards integrating LLMs into these platforms and workflows. The literature recommendation task is well-suited to automation, as a moderate rate of false positives or false negatives does not significantly impact overall effectiveness. Additional protocols can ensure comprehensive document retrieval, while triage can quickly filter false positives. The existing tools and datasets offer a critical foundation for more advanced, AI-based curator-in-the-loop systems that enhance efficiency and accuracy across the biocuration pipeline.

### 2.2 Named entity recognition

With literature triage increasingly aided by AI, the next challenge lies in accurately identifying the biological objects mentioned in texts, laying the foundation for downstream curation. NER is an information extraction subtask that identifies and classifies key annotatable entities within text. While the term ‘entity’ can broadly encompass annotated data types, such as functions, processes, modifications, and phenotypes, in this discussion, we restrict its meaning to refer specifically to annotated biological objects, including genes, proteins, and related biomolecules. In text mining pipelines, NER is often followed by two further related subtasks: ‘normalization’ and ‘grounding’. Normalization refers to mapping various synonymous expressions of an entity to a standardized form (e.g. mapping gene names ‘p53’ and ‘LFS1’ to the canonical name ‘TP53’). Grounding, also known as ‘entity linking’, involves linking these normalized entities to unique identifiers in authoritative databases, such as the NCBI Taxonomy Database or UniProtKB. These steps ensure consistent and unambiguous reference to biological entities across datasets and tools.

#### 2.2.1 Tools and resources for named entity recognition

*Europe PMC* (europepmc.org) employs text mining to identify biomedical entities within the text, including species names, genes or proteins, diseases, GO terms, metagenomic terms, chemicals and molecules, experimental methods, and gene–disease relations. These entities are grounded to a corresponding unique entry identifier in the relevant databases or ontologies and are available in the ‘Data Links’ section or via the SciLite annotation panel (Venkatesan *et al.* 2016, Tirunagari *et al.* 2025). Other text-mining groups can participate in the Europe PMC Annotation platform by submitting their annotations.

*GNorm2* (github.com/ncbi/GNorm2) is a key tool integrated into PubTator that uses transformer-based deep learning methods for gene name recognition and normalization tasks (Wei *et al.* 2023). Two language models are available: PubMedBERT, for higher accuracy (Gu *et al.* 2022), and Bioformer, for increased speed (Fang *et al.* 2023). A species recognition and assignment module is also available (Luo *et al.* 2022). This tool reports high accuracy and efficiency for gene recognition and normalization with an *F*_1_-score (the harmonic mean of precision and recall) of 0.894 using PubMedBERT for NER. GNorm2 is being integrated into the curator workflow at the National Library of Medicine to increase efficiency in manual gene linking (Islamaj *et al.* 2025). The NCBI plans to apply GNorm2 to the entire content of PubMed and PMC to support downstream biomedical NLP research.

*Pre-publication entity recognition.* Comprehensive NER prior to publication remains uncommon, but *microPublication Biology*, a platform dedicated to publishing brief, data-driven reports that might otherwise go unpublished, has integrated pre-publication resolution of gene and allele entities into its editorial workflow (Raciti *et al.* 2018). This integration is achieved by combining author-driven curation with database partner validation. As part of the article submission process, authors specify the species and data types of interest, which are then recorded in the publication’s metadata. A linking tool, powered by species-specific dictionaries provided by partner databases, highlights known entities for curator review and verification. This pipeline flags potential errors and ambiguities, enhancing naming consistency and standardization.

NER presents several common challenges for LLMs, including difficulties in identifying gene names that are common English words, dealing with the non-universality of nomenclature, and handling multiple species in a single publication (Wei *et al.* 2023). So far, species-specific dictionaries have proven necessary to avoid false positives when recognizing biological entities in a production setting at *microPublication Biology* (Karen Yook, personal communication, 14 May 2025), and are routinely used by knowledgebases. However, early RAG approaches are encouraging when grounded using curated reference repositories, substantially improving gene and protein mention detection and normalization. For example, the Alliance for Genome Resources is piloting RAG-powered LLMs on full-text publications for complex NER and has even shown promising results with unseen *Drosophila melanogaster* transgenic constructs (Christopher J. Tabone, pers. comm., 05 May 2025, unpublished work).

### 2.3 Approaches for data type identification and relation extraction

Determining which information is curatable and how it supports structured associations between biological entities and ontology terms is one of the most complex and time-intensive aspects of biocuration. This task encompasses multiple data types, including GO terms, phenotypes, diseases, modifications, and interactions (see Fig. 2 for examples). Effective fact-extraction approaches must map extracted statements to the appropriate ontology terms and provide the text span used, enabling curators to verify the result.

Numerous model organism knowledgebases and the GO Consortium report that agentic approaches are showing promising results in first-pass curation. Although no formal case studies have been published, these methods can deliver outputs aligned with established curation standards, including conversion of the extracted facts into ontology terms, providing the extracted text for manual review and associating the extracted term with appropriate evidence codes. This approach has the potential to increase the speed of manual curation while maintaining context and accuracy. The use of LLMs for data extraction is still emerging; here, we present examples of state-of-the-art tools currently in use.

*EnzChemRED* (Enzyme Chemistry Relation Extraction Dataset) provides a curated dataset of 1210 PubMed abstracts to train and benchmark NLP tools for enzyme curation (Lai *et al.* 2024). Fine-tuning pre-trained LMs using the EnzChemRED dataset significantly improved the extraction of enzyme entities (chemicals and proteins) (*F*_1_ 0.863), and chemical reactions (*F*_1_ 0.837). These fine-tuned models were then applied at scale to identify enzyme activities in the literature, supporting manual curation efforts for the UniProtKB and Rhea databases.

*FuncFetch* is an LLM-assisted workflow that integrates NCBI E-Utilities and OpenAI’s GPT-4 to screen thousands of manuscripts and extract enzyme activities and substrates (Smith *et al.* 2024). It retrieves species information, enzyme names, sequence identifiers, substrates, and products. When benchmarked against a manually curated dataset of BAHD family acyltransferase activities, FuncFetch achieved a precision of 0.86 and a recall of 0.64 in substrate extraction.

*GOFlowLLM* is a reasoning-enabled curation extraction pipeline that leverages LLMs to follow established GO curation flowcharts for miRNA curation and to extract miRNA–protein interaction data at scale (Green *et al.* 2026). Applied to 6996 previously uncurated articles, GO-Flow identified 1826 new candidate GO annotations in just 56.7 h. Manual evaluation of a representative subset showed that curators agreed with the selected GO term in 86.7% of cases, judged the model’s reasoning sound in 91.7%, and found the extracted evidence adequate in 93.3%. GO-Flow extracts supporting text directly from publications and presents curators with both the evidence and the model’s reasoning. This approach addresses a key challenge by accelerating biocuration while preserving expert review and evidence traces.

Despite recent innovations, the performance of data extraction methods varies across domains and data types. Data types with relatively low semantic variability, such as physical interactions, GO cellular components, and chemistry concepts (including enzymes and reactions), have already shown promising results. In contrast, there are currently no published examples of extracting detailed, standardized phenotypes, complex biological processes, and causal relationships between entities supported by appropriate evidence.

Methods that rely on training data require large amounts of carefully curated, gold-standard data. This approach becomes ineffective when dealing with new or emerging knowledge, where such data are scarce or nonexistent—the very gaps that need to be filled—and where hallucinations are more likely to occur. The introduction of instruction-tuned LLMs provides an alternative approach, in which the LLM is provided with human-readable documentation stored in a vector database and the target publication. It uses RAG to determine the most effective method for extracting the required data. Refining these instructions is far more tractable than creating labelled datasets for every application. Alongside RAG to retrieve the guidelines, modern LLMs can apply reasoning effort to solve problems. This approach enables an LLM to determine what experimental evidence is required to make annotations, then reason about how the evidence in an article aligns with those requirements to select the most appropriate annotation. Such agentic approaches are potentially transformative because they can present the precise source text used to make an association to fact-checkers, thereby speeding up the curation process.

The model organism knowledgebase community is actively testing agentic AI systems. These systems can retrieve information and perform initial verification (self-checking) by cross-referencing multiple sources. They can present human curators with a pre-validated set of entities associated with ontology terms, along with supporting evidence and traceability. These advances should significantly reduce the manual workload while making the entire curation process explicit and reproducible through revisible rules. Although such systems are being evaluated at several knowledgebases (including FlyBase, the AGR, and the GO), no results have yet been published. The main challenge ahead will be to build specialized, task-specific agents and the capacity to evaluate AI-generated outputs.

### 2.4 Knowledge summarization

Knowledge summaries are concise textual overviews that capture key biological insights for specific entities. These summaries help researchers, especially those who are not experts in the gene, to quickly grasp the most relevant information from a vast body of curated data. For example, the *cdc2* gene page in PomBase includes over 600 distinct entity-term associations encompassing multiple data types. When confirmatory associations supporting reproducibility are included, this number rises to more than 1680. While this level of detail is a rich reference for experts, it can overwhelm users attempting to interpret large-scale results. Many databases address this by providing manually crafted summaries synthesized from curated data or reviews. However, such summaries are time-intensive to create, burdensome to maintain, and often inconsistent in format, making automated summarization an attractive alternative. WormBase pioneered automated gene summarization using rule-based methods and ontology-based curation (Kishore *et al.* 2020). More recently, numerous strategies have successfully applied LLMs to literature summarization tasks.

*InterPro* (www.ebi.ac.uk/interpro) has successfully adopted LLMs to automate the description of protein families (Blum *et al.* 2025). Traditionally, when protein families are integrated into InterPro, curators have to review the scientific literature and write manual descriptions. InterPro now streamlines this process using LLMs. It leverages high-quality, existing curation from Swiss-Prot entries and creates a structured prompt for the LLM. This prompt instructs the model to generate a summary of the protein family’s function and key characteristics. As of version 99.0 (March 2024), InterPro contained over 3000 AI-generated summaries.

*LitSumm*—RNAcentral has similarly adopted an LLM-based approach, LitSumm, to summarize literature related to non-coding RNAs (Green *et al.* 2025). The LitScan text mining tool searches the Europe PMC API for candidate non-coding RNA identifiers and extracts sentences containing identifiers from over 177 000 articles. Topic modelling is conducted through sentence transformers, which convert sentences into vectors and cluster them. GPT-4 then processes the clustered sentences using a set of curated prompts designed to minimize hallucinations and ensure accurate, unbiased information supported by appropriate references. The LLM checks each summary against its source sentences, with pass rates rising from about 85% on the first attempt to 97% after up to four rewrites. Over 4600 ncRNA summaries are available via the RNAcentral resource.

LLMs are uniquely suited to gene summarization tasks because their pre-training on vast natural language corpora enables them to process and contextualize complex biomedical literature at scale. Their contextual understanding, information synthesis, and summarization capabilities allow them to generate accurate, consistent, and up-to-date summaries that complement or improve manually written summaries. LLMs’ instruction-following capabilities simplify the development and optimization of summarization pipelines, eliminating the need for model fine-tuning and allowing improvements to be made simply by refining the instructions. These capabilities position LLMs as powerful tools for scalable and consistently current summaries of collected knowledge.

### 2.5 Ontology development

Ontologies are essential to biocuration, providing a structured and computable representation of consensus biological knowledge. However, developing and maintaining bio-ontologies is a resource-intensive process. Curators are responsible for proposing, defining, and often implementing new terms, as well as correcting legacy terms, adding to their already substantial workloads. Streamlining ontology development can directly reduce the burden on curators and improve overall curation efficiency.

*Dynamic Retrieval-Augmented Generation of Ontologies using AI* (DRAGON-AI), developed by (Toro *et al.*2024), combines LLMs with RAG to support ontology construction. DRAGON-AI can generate both human-readable definitions and formal logical axioms for new terms using existing ontologies and unstructured text sources. Ontology editors evaluated its performance in creating new terms across ten diverse ontologies through expert manual review of term definitions and logical structures. Notably, this study found that more experienced curators were better at identifying flaws or inconsistencies in the generated definitions, highlighting the importance of curator expertise in ensuring ontology quality and AI content more generally.

The DRAGON-AI and related approaches can significantly accelerate ontology development, and the GO ontology editors already use an extended agentic approach in production to perform an increasing number of routine but laborious ontology development tasks.

### 2.6 Curation and quality control assistants

Many biocurators now use LLMs interactively as assistants to support real-time decision-making, such as locating source material, checking knowledge about unfamiliar genes or pathways, or assessing the biology of lesser-known species. As discussed above, agentic approaches are being actively evaluated to provide standardized first-pass fact extraction. In addition to supporting general queries and new curation, agentic-LLMs show strong potential for retrospective quality control. They can augment curator workflows by automating routine checks, identifying semantic or logical inconsistencies, verifying reasoning traces, flagging deviations from curation standards, and enhancing consistency across knowledgebase contents without replacing human expertise.

*Paper-QA2* (futurehouse.org/research-announcements/wikicrow) is a general-purpose, LLM-based system for querying scientific literature (Skarlinski *et al.* 2024). It supports interactive use with one or more documents, handles custom queries, and generates context-aware responses grounded in the source material. By performing multi-step, chained reasoning to identify gaps and guide subsequent actions, it illustrates how such tools can assist curators in navigating and synthesizing scientific information.

*Aurelian* (Agentic Universal Research Engine for Literature Integration, Annotation, and Navigation) is a command-line suite of task-specific AI agents designed to assist human curators (Mungall *et al.* 2025). Agents include support for creating and reviewing GO associations, creating GO-Causal Activity Models (GO-CAMs), ontology mapping, rare disease diagnosis, and chemical structure analysis.

LLM-based assistants and emerging agentic systems are increasingly supporting biocurators in ensuring data quality by streamlining evidence retrieval, fact verification, and other QC-critical tasks, exemplified by tools like Paper-QA2 and Aurelian.

## 3 Challenges and recommendations

The widespread integration of AI into biocuration workflows presents both substantial opportunities and significant technical, organizational, and conceptual challenges. The following subsections outline critical areas that must be addressed to ensure the robustness, scalability and utility of AI-assisted curation. These include the need to assess which tasks are suitable for automation, the importance of generating high-quality training data, the development of rigorous benchmarking frameworks for evaluation, and the value of sustained collaboration to avoid duplication and maximize reuse. Human-in-the-loop oversight is essential at every stage of the AI literature-curation workflow to ensure continuous feedback and informed recommendations. The recommendations presented here synthesize observations from ELIXIR CDRs and GBCRs, reflecting both community-wide priorities and resource-specific considerations. They are intended to guide the systematic integration of evidence-based AI into literature-curation pipelines, while maintaining the scientific integrity of curated knowledge.

### 3.1 Suitability, value, and sustainability

A common perception, particularly outside the biocuration community, is that biocuration tasks can be largely automated. A more realistic assessment is needed to identify which biocuration contexts are genuinely suitable for AI support, and to acknowledge that substantial human curator input will remain essential. While many specific, well-defined tasks show promise for AI-based solutions, the technology’s most impactful role is as an intelligent assistant, augmenting curator workflows with task-specific support.

Full integration of AI into biocuration workflows requires a phased approach that carefully manages the value proposition. The initial costs involve significant human investment, as curators must create and iteratively refine AI instructions as well as provide gold-standard data for training and evaluation. The gains, however, can be substantial and enduring. By leveraging AI to automate repetitive, well-defined tasks, human curators can focus on complex reasoning, interpretation, and quality control, thereby dramatically increasing overall efficiency and output. A careful transition is crucial to maintain the continuity of the existing biocuration workforce and prevent the loss of critical institutional knowledge.

A prime example of a high-gain, low-cost opportunity is a task that remains largely unaddressed: estimating the total number of curatable publications, identifying those already curated, and determining their data types. This analysis would provide biological resources and their funders with a clear view of the total human effort required for comprehensive curation, pinpointing precisely where targeted AI support would offer the greatest return on investment. This foundational work would help prioritize tasks and allocate resources where AI can deliver the greatest value.

#### 3.1.1 Sustainability

A key concern, considering the extraordinary pace of change in this field, is the human, financial and environmental burden of sustaining and adapting existing AI pipelines as agentic systems continue to evolve; Perhaps counterintuitively, the development cost of an agentic system is mainly in its evaluation, which is still completely reliant on human rating of agentic outputs. The development of the software underlying the agent is relatively inexpensive and can, increasingly, be offloaded to the agent itself. By providing a universal interface to tools the Model Context Protocol (MCP) enables LLMs to be largely interchangeable, meaning the same set of tools and prompts can be reused when a model is replaced or updated. Most of the maintenance burden lies in evaluating new agentic systems enabled by improved models.

Strategies that reduce computing requirements and lower energy consumption should always be a consideration. The environmental impact can be lessened by prioritizing the use of smaller, biologically-focused models over general-purpose ones, or developing compact models that replicate the performance of larger models wherever possible, and running models locally or on energy-efficient hardware whenever feasible.

### 3.2 Training and evaluation

#### 3.2.1 Training data and grounding corpora

Harnessing AI to support functional curation from hypothesis-driven experiments currently requires access to high-quality, domain-specific training data, or, in the case of RAG approaches, grounding resources (e.g. authoritative named entity lists). One key limitation is the scarcity of computationally accessible gold-standard datasets that capture the information curators use in decision-making. These data can include sentence-level publication text spans or figure references connected to the final curated associations. Valuable training data already exists, but it is widely dispersed and difficult to locate. Consolidating potential training datasets into a unified, well-documented platform could accelerate AI development for biocuration. It may also be feasible to ‘reverse engineer’ sources of evidence from expert-curated data, using the methods described in the GO-Flow project, allowing the more rapid assembly of richer and more detailed training data.

Curated KGs offer a structured, semantically rich representation of biological knowledge. They establish explicit relationships between entities, enabling systems to reduce ambiguity and enhance prediction accuracy. Unlike computationally generated KGs, which are often shallow or error-prone, expert-curated KGs encode verified and precise domain-specific knowledge.

Many biocuration resources already maintain connections between biological entities. For example, UniProtKB includes over 210 billion subject–predicate–object relationships modelled as RDF triples (although only a subset of these connect biological entities) (sparql.uniprot.org), and GO annotations include 48 000 causal entity-to-entity connections using annotation extensions—a framework introduced by the GO Consortium to enrich standard annotations by specifying effector–target relationships and providing additional biological context to curated associations (Huntley *et al.* 2014). More detailed causal models are provided by GO-CAMs and Reactome pathway curation (Gillespie *et al.* 2022). For instance, GO-CAM integrates multiple standard GO annotations into network-like models that represent how gene product functions are coordinated in biological systems. These curated models, along with those from Reactome, form an ideal baseline of communal prior structured knowledge for training more robust and accurate AI systems (Dessimoz and Thomas 2024).

#### 3.2.2 Benchmarking and evaluation

AI-assisted biocuration systems must be evaluated using transparent, domain-specific benchmarks or human evaluation to ensure reliability and reproducibility. Evaluation methods may include measuring precision and recall using manually curated test sets, inter-curator agreement scores, or robustness testing through controlled data perturbations. Feedback loops are also critical: tracking AI outputs after expert review can guide model retraining, prompt refinement, or fine-tuning. Community benchmarks and clear guidelines are needed to support consistent evaluation mechanisms across projects. While existing standards such as the DOME guidelines (dome-ml.org/guidelines) and recent proposals (Farrell *et al.* 2025) provide a foundation for describing AI models, biocuration workflows also require domain-specific criteria that address traceability, explainability, adherence to curation guidelines, and demonstrable improvement in curation quality.

The need for guidelines and expert evaluation was recently highlighted by De Crécy *et al.* who reviewed a controversial study that employed deep learning to predict complete Enzyme Commission (EC) numbers for 295 previously uncharacterized *Escherichia coli* enzymes (Kim *et al.* 2023, de Crécy-Lagard *et al.* 2025). They showed that approximately 30% of the predicted functions reported were already annotated in UniProt, while an additional 60% could be clearly refuted by experts based on existing literature or biological context.

Evaluation remains a critical step in AI-assisted curation; however, it is often a bottleneck due to its dependence on already overburdened domain experts and curators. Historically, evaluation has been a low-tech process, with curators frequently reviewing AI-generated outputs manually in spreadsheets. Meanwhile, the broader machine learning community has developed highly configurable open-source data curation and feedback tools for NLP and LLM workflows—such as Argilla (argilla.io) that can streamline and accelerate this review process. Such tools can be used to present reasoning traces to support decisions, or to present text spans containing the experimental evidence that support associations, allowing them to be rapidly reviewed by curators. In the GO-flow project, this method allowed curators to make informed decisions about the extracted GO term and the supporting experimental evidence without needing to consult the original article in most cases. The estimated time reduction was around 80% for this relatively straightforward curation task. If the evaluation process is sufficiently streamlined and if both accuracy and recall are sufficiently high, it could also be feasible to engage publication authors in assessing AI-based fact extraction for their own articles using this mechanism.

International community challenges such as BioCreative and CAFA have been instrumental in evaluating and advancing text-mining and AI-based biocuration methods (Wang *et al.* 2016, Zhou *et al.* 2019). These challenges provide shared datasets and objective evaluation metrics, and foster collaboration between developers and curators. Expanded benchmarking efforts that are focused on AI-driven fact extraction and knowledge integration could be highly valuable. Additional BioCreative-style challenges could play a key role in maintaining methodological rigour, ensuring reproducibility, and guiding the development of reliable, production-ready AI tools to support literature-curation tasks. In addition, the biocuration community could support the AI community by routinely providing datasets that capture the text spans and figure numbers curators use to make assertions.

A robust approach to benchmarking AI-based literature-curation systems requires comprehensive evaluation across precision and recall. Systematically collecting rejected AI suggestions will improve false-positive quantification and identify systematic failure modes, while simultaneously tracking human-added entities (that the AI missed) to measure false negative rates. Accepted AI suggestions should be classified by association type, distinguishing between previously annotated, newly identified, and genuinely unknown associations, to assess performance across different knowledge subdomains. This framework would move beyond simple accuracy metrics to provide actionable insights into AI behaviour based on the decisions of human evaluators (retained and rejected entities and associations). Such a continuous feedback loop provides the foundation for incremental learning approaches that can improve model performance by learning from real-world curation decisions (rather than static datasets).

Achieving the benefits of AI-assisted curation will require robust benchmarking frameworks and sustained oversight by a human-in-the-loop to prevent errors from being introduced or propagated. In the near term, limited curator capacity will remain a significant bottleneck. However, as benchmarking and evaluation methods advance, agents may increasingly handle end-to-end biocuration tasks, supervised by human curators. In this paradigm, LLMs act as force multipliers, accelerating annotation workflows while curator-reviewed high-quality data improves AI performance in return.

### 3.3 Ensuring accuracy

There are legitimate concerns about the introduction and propagation of AI-derived errors within and across biological databases, which can lead to wasted resources (Pop *et al.* 2025). These risks mirror a broader challenge: scientific knowledge itself is never static. Biology evolves through reinterpretation of experimental results as new methods and insights emerge. Hypotheses about gene function are often revised—or overturned—based on more accurate data. For example, TCF25 was initially described as a transcription factor but has recently been discovered to be involved in ribosome quality control (Dos Santos *et al.* 2024). Similarly, WHR1 (previously known as STK19), once considered a kinase implicated in Ras-mediated signalling, has no kinase activity and is now known to function in transcription-coupled DNA repair (Li *et al.* 2024). For nearly a generation, the yeast TTT complex was regarded as a chromatin-remodelling complex (ASTRA). However, it was recently demonstrated biochemically to be an Hsp90 cochaperone that promotes cotranslational maturation of a family of inositol kinases before complex assembly (Toullec *et al.* 2021). Such paradigm shifts permeate the literature, highlighting the vital role of knowledgebases in capturing and updating the collective understanding so that outdated interpretations do not persist. Nevertheless, legacy findings persist in LLMs because older literature often outweighs newer corrections.

Preventing the reintroduction of superseded or disputed associations requires robust mechanisms to identify and retire associations no longer supported by more recent publications. Recognizing these risks, the biocuration community maintains strict oversight. Currently, within the GBC-GBCRs or ELIXIR CDRs, there are no known examples of AI-augmented data extraction being incorporated without human oversight. For instance, GO guidelines require that a trained curator manually review AI-derived gene-to-term associations from publications to verify experimental evidence, accuracy, and compliance with GO standards before they are accepted as standard experimental gene associations.

Agentic AI systems can support error mitigation in data extraction by combining goal-directed reasoning, task decomposition, tool use, and iterative feedback loops. A central aspect of this process is the agent’s ability to access, query, and verify external knowledge sources—including publications, ontologies, and specialist databases—whenever additional context is required. By retrieving and cross-checking information during a workflow, agents can reduce hallucinations, improve attribution, and maintain grounding in sparse or specialized domains.

Modern agentic approaches routinely delegate subtasks such as information retrieval, database queries, validation, and composition to specialized tools. Although this improves robustness, autonomy also introduces new failure modes: agents may choose suboptimal tools, enter unproductive loops, or combine steps in unexpected ways that propagate reasoning errors. A critical enabler of reliable agentic AI is therefore the integration of well-designed, discoverable tools. The Model Context Protocol (MCP) generalizes this principle by providing a universal interface to tools, including search endpoints, databases, or analytical services, so that agents can automatically discover, invoke, and coordinate them as part of multi-step plans. MCP servers already exist for many components of literature curation, including Europe PMC and the Ontology Lookup Service. To ensure agents remain grounded in the latest knowledge, we recommend that resources provide MCP servers for their tools, register them in platforms such as BioContextAI, and iteratively refine their design and functionality in collaboration with AI-agent builders.

Another promising safeguard is leveraging AI systems to audit and refine each other’s outputs. This approach—sometimes referred to as *AI-on-AI validation*—utilizes multiple, independent models or pipelines to cross-examine results before they reach human curators. Combined with expert oversight, RAG and agentic AI provide a powerful way to detect errors and outdated content, ensuring modern databases stay accurate while auditing, refreshing, and expanding both new and legacy curation efforts as scientific knowledge evolves.

### 3.4 Collaboration

Collaboration between and within the biocuration and AI communities is critical to realizing the full potential of AI-assisted curation workflows. The biocuration field has long maintained a collaborative ethos, developing shared standards, minimizing redundant effort, and coordinating across resources. Extending these practices to create robust, reusable AI solutions is essential given the severe resource constraints.

Close collaboration could accelerate progress in several areas, including standardizing metadata tags in literature pipelines, developing standards for describing gold-standard training datasets, establishing common mechanisms to flag AI-generated outputs in curated resources, and identifying domain-specific expert evaluators. To support transparency and reproducibility, training data, evaluation frameworks and prompting strategies should be openly shared and reused where possible.

By integrating domain expertise with AI specialists’ knowledge, teams can avoid wasted effort, as the domain experts ensure proposed solutions are practical and likely to succeed in real-world applications.

## 4 Conclusions

The transformative potential of AI in biology, exemplified by breakthroughs like AlphaFold, depends on high-quality biological data and on rigorous assessment frameworks, such as those provided by CASP. Just as AlphaFold relied on high-quality structural data, the next generation of AI-driven discoveries in functional biology will depend on well-curated knowledgebases, such as pathways and structured data, rather than on raw experimental data alone (Dessimoz and Thomas 2024). In particular, we envisage that curated causal models and KGs will provide a gold-standard ground truth for training and evaluating LLMs.

This perspective shows that AI integration in biological knowledgebases has progressed from isolated prototypes to operational use within active curation workflows. Sustained progress will require standardized evaluation protocols, openly shared benchmark datasets, and coordinated development within collaborative biocuration frameworks.

Paradoxically, this need arises amid declining funding for biocuration and growing demand for comprehensive, high-quality datasets (Kaiser 2016, Oliver *et al.* 2016, Dessimoz and Thomas 2024). Knowledgebases underpin the interpretation of experiments and guide future research; errors can propagate with serious consequences (Pop *et al.* 2025). Supporting the human expertise that underpins these resources is therefore essential.

AI can amplify curator efforts by automating well-defined, repetitive tasks, allowing experts to focus on complex reasoning, interpretation, and quality control. Safeguards such as RAG, agentic AI, and AI-on-AI validation can further ensure accuracy, enabling models to cross-check outputs and flag outdated or incorrect information before it contaminates curated resources.

### 4.1 Human-in-the-loop

**Figure 3:**
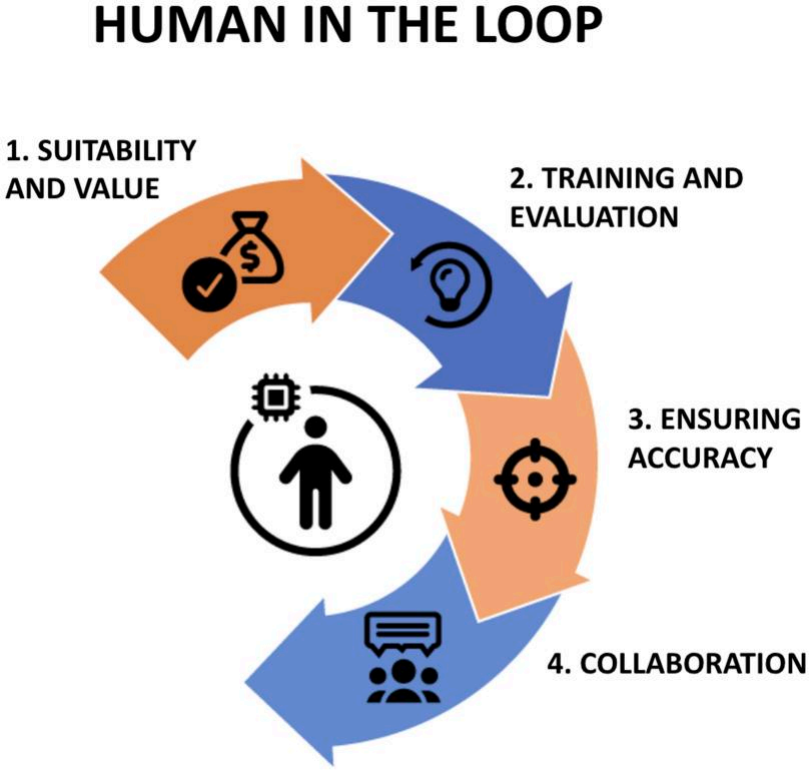
A schematic view of human-in-the-loop for AI-augmented biocuration. A human curator guides AI-driven workflows across four key areas: (1) Suitability & value: identify tasks suitable for automation and prioritize high-value curated datasets. (2) Training & evaluation: provide high-quality training data and benchmarks; ensure reliable model performance. (3) Ensuring accuracy: oversee AI outputs; use safeguards like RAG and AI-on-AI validation to prevent errors. (4) Collaboration: promote shared standards, interoperable datasets, and community-driven evaluation for reproducibility and accelerated discovery. Human oversight creates a virtuous cycle: AI accelerates curation, and the curated data in turn improves AI performance.

By interpreting published findings and integrating them into ontologies and databases, biocurators have historically enabled the formation of consensus knowledge and the emergence of new insights. These longstanding contributions will continue to be indispensable, even as AI changes the nature of biocuration. In the evolving AI-enabled landscape, biocurators will additionally identify tasks suitable for automation, generate high-quality training data and benchmarks, provide evaluation, refine outputs, and ensure that results are biologically meaningful and robust (Fig. 3).

This human-in-the-loop framework creates a virtuous cycle—AI accelerates curation, curators provide evaluation, and curated data improves AI performance—driving continuous improvement and robust knowledge management. However, securing the resources required to manage this transition is a challenge, particularly in light of the substantial additional burden placed on curators by being the human-in-the-loop. As a result, many organizations adopting these systems expect a temporary reduction in productivity as AI-driven tools are evaluated and implemented.

Collaboration across the AI and biocuration communities will amplify these gains. Shared standards, interoperable datasets, and open evaluation frameworks will minimize redundancy, enhance reproducibility, and accelerate progress. Cross-domain expertise will allow iterative refinement, ensuring AI outputs are both practically valid and scientifically reliable.

AI will deliver scalable computation and speed, while humans provide critical judgement, contextual insight, and ethical oversight. Together, this synergy will enable knowledge systems that not only keep pace with the rapid evolution of biological science, but actively *accelerate* discovery, transforming the future of functional biology.

## Data Availability

This article is a review and does not include any original data. No new data were generated or analysed in this study.
